# A Baton for Karaoke Intertwined with The Blade of The Surgeon

**DOI:** 10.1055/a-2688-4145

**Published:** 2025-09-18

**Authors:** Joon Pio Hong, Marco Innocenti, Geoffrey G. Hallock

**Affiliations:** 1 Department of Plastic and Reconstructive Surgery, Asan Medical Center, University of Ulsan College of Medicine, Seoul, Republic of Korea; 2 Orthoplastic Surgery Department, Rizzoli Institute, University of Bologna, Bologna, Italy; 3 Division of Plastic Surgery, St. Luke's Hospital, Sacred Heart Division, Allentown, Pennsylvania

**Figure:**
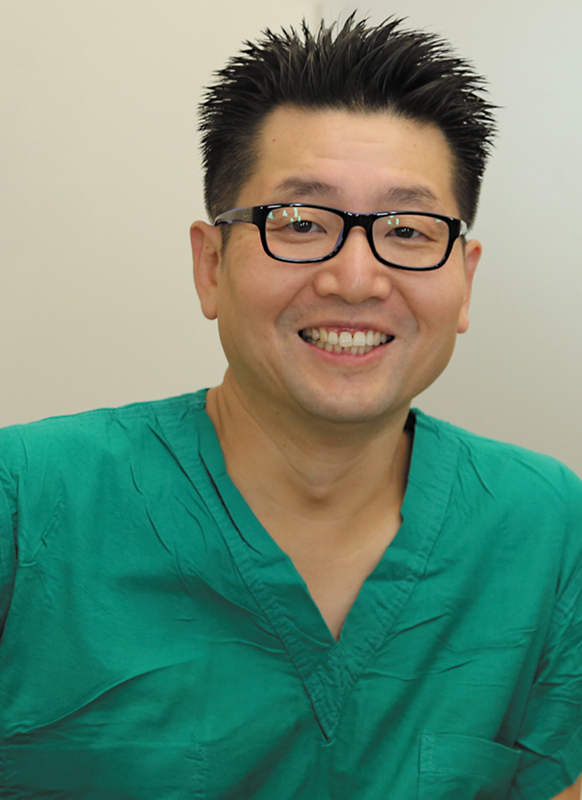
Joon Pio Hong, MD, PhD, MMM (Editor Emeritus of APS)

**Figure:**
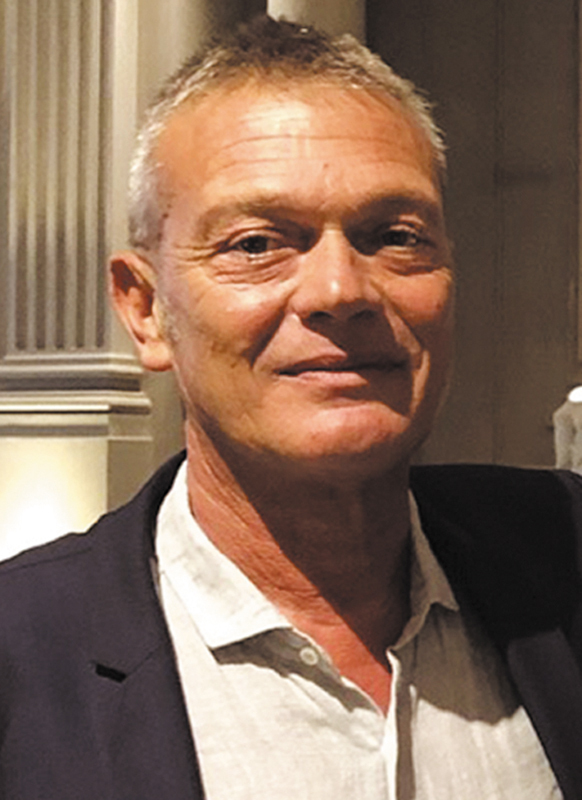
Marco Innocenti, MD

**Figure:**
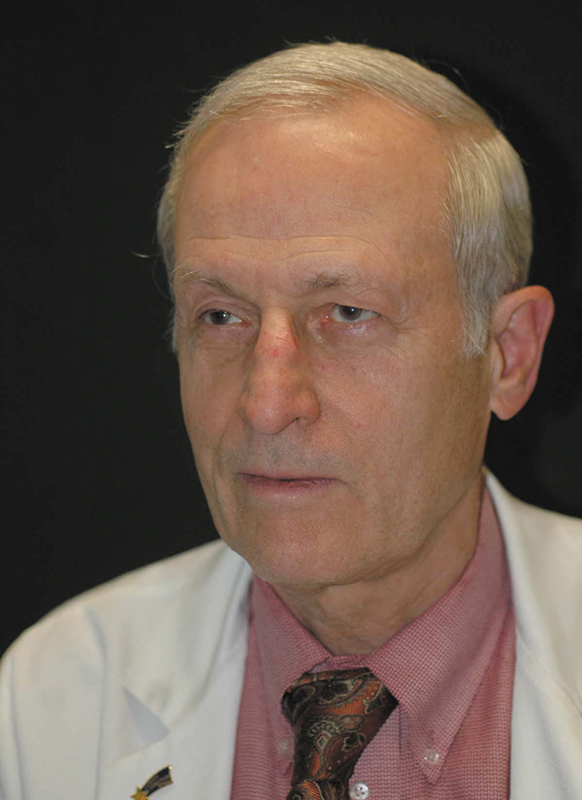
Geoffrey G. Hallock, MD

History we are. Indeed, we know Greek πλαστική ( *plastikē* ) refers to the art of modelling or sculpting, especially in clay. 1 Understandably then, “plastic surgery” as conceived by Zeis (1938), 2 is an art where inert materials have been replaced by more malleable flesh. 1 Can it be a mere coincidence that the etymological tree leading to “karaoke” (some say ker-ē-ˈō-kē, others kə-ˈrō-kē or kä-rä-ˈō-(ˌ)kā), 3 also has roots in ancient Greek ὀρχήστρα ( *orkhḗstra* ), this leading to the now universal English (orchestra). 3 4

Yet the transition to the word “karaoke” itself must be credited to the Japanese just a half-century or so ago as a blend of their words kara (“empty”) and オケ ( *oke* ), a clipping of オーケストラ ( *ōkesutora* ) as derived from the English “orchestra.” 4 So-called “empty” because no musical instruments typically are present and those involved sing-along to a prerecorded accompaniment that projects their voice and comments. 3 5 “Empty” may also be interpreted as a void that must be filled by some amateur and often vulnerable performer(s) (as per Fig. 1 ).

**Fig. 1:**
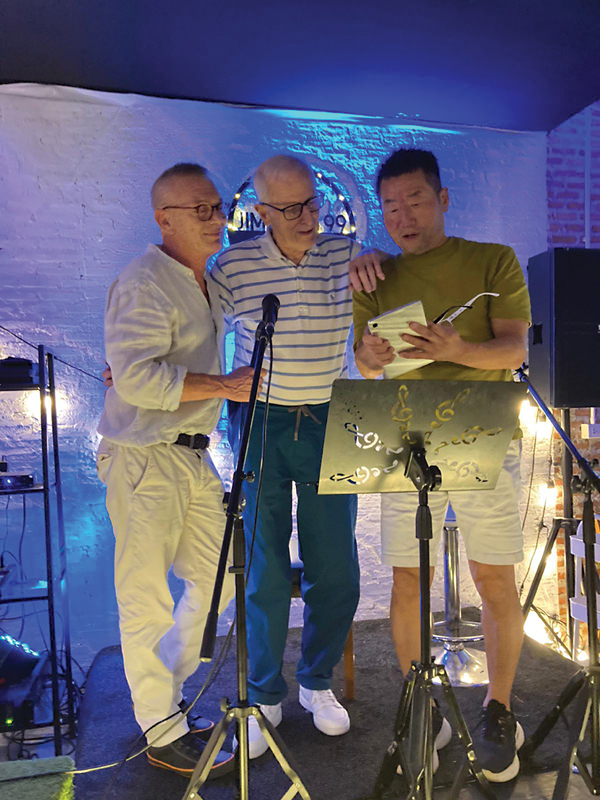
*Les Trois Mousquetaires*^6^*—* The Three Musketeers leading the “karaoke”—Innocenti, Hallock, Hong—in turn *heroic* , *chivalrous* , and always *swashbucklers* .

Not to forget Daisuke Inoue, some say the inventor of karaoke, the winner of the 2004 Ig (sic Ignoble) Nobel Peace Prize, who in satirical fashion retorted that the essence of his invention was “providing an entirely new way for people to learn to tolerate each other.” 7 A less contemptuous tone might say that karaoke is just another form of music, and for some physicians, another medicine. Do not forget that even the ancient Egyptians and Greeks used music to control the mood and physiological responses of their patients. 8 The efficacy of music both preoperatively and postoperatively in patient management has a long and documented history. 9 10

On the other side of the coin, music in the operating theatre itself can enhance the efficiency of the performance of microsurgical skills, 11 12 13 by reducing stress, providing relaxation, and promoting concentration. 14 15 Even behold, already there is found music, 8 where in the future we all will use while performing robotic surgery. 16 This intimate connection to surgeons should not be so surprising since a host of cognitive skills are common to both music and surgery—intensity of concentration, advance anticipation of all steps to follow, strictness in adhering to the proper order, improvisation or flexibility to overcome the unexpected event, virtuosity of a master who can spontaneously and effortlessly adapt as need, an ability to listen to the other members of the team and understand their concerns, while creating harmony so all together achieve the desirable outcome. 14 Music better teaches how to listen, a step essential in learning empathy and compassion, 14 17 virtues rarely intertwined in surgeons but deserve to be. 18

Yet some say karaoke is more than just the music itself. Metaphorically speaking, karaoke is a form of schooling and education, and not just entertainment. 19 Whereas traditional schooling has norms of behavior that must be abided by, karaoke is basically free of constraints so that the learning is never premediated but more improvisational, perhaps even innovational. 19 Innovation, not dogma, has always been on the tip of the tongues of these three musketeers. 20

Where does this all lead us? The emphasis today is to seek “happiness” and avoid burnout by achieving the now cliché of “work–life balance.” 21 22 23 24 25 Some might camouflage this as “work–home” 23 or even “work–leisure” balance, 25 but no matter not always so simple a dichotomy. Should not our professional duties be better if integrated to encompass our lives outside the workplace? 24 Opportunities exist in both venues to build rewarding relationships and accomplishments that create an even greater impact concurrently for the lives of our patients, our teammates, and ultimately ourselves. A simple means to calmly achieve this goal, these same three musketeers have found with just “karaoke”—simultaneously allowing us to grasp networking and leisure with our peers, for always an improvement.

So remember when Rohrich questioned—“What do you do when the music stops? … You keep dancing.” 26 Life always has surprises—be they unexpected detriments, obstructions, mere roadblocks, the daily new rules and instructions, and sometimes even administrators that will need to be overcome. As surgeons, we must rely on our past and present preparation and skills to conquer whatever may be the confrontation in all aspects of life. So, “What do you do when the karaoke stops? …You keep operating!”

## References

[1] Britannica. “Plastic surgery.” OED Online. Accessed June 15, 2025. Available at :https://www.britannica.com/science/plastic-surgery

[2] Zeis E Handbuch der plastischen Chirurgie Berlin Reimer 1838

[3] “Karaoke” Merriam-Webster dictionary. Accessed June 15, 2025. Available at:https://www.merriam-webster.com/dictionary/karaoke

[4] Wiktionary.カラオケ#Japanese:_karaokeAccessed June 15, 2025 at:https://en.wiktionary.org/wiki/

[5] Wikipedia. Karaoke. Accessed June 15, 2025. Available at:https://www.wikipedia.org/karaoke

[6] Wikipedia.The Three MusketeersAccessed June 15, 2025 at:https://en.wikipedia.org/wiki/The_Three_Musketeers

[7] Improbable Research.“The 2004 Ig Nobel Prize Winners”August2006. Accessed June 15, 2025. Available at:https://www.improbable.com/ig/winners#ig2004/

[8] Siu K C Suh I H Mukherjee M Oleynikov D Stergiou N The effect of music on robot-assisted laparoscopic surgical performance Surg Innov 2010 17 04 306 311 20817638 10.1177/1553350610381087

[9] Zapata-Copete J A Cordoba-Wagner M J García-Perdomo H A Role of music in a plastic surgery setting: A systematic review and meta-analysis Indian J Plast Surg 2019 52 02 160 165 31602130 10.1055/s-0039-1696792 PMC6785311

[10] Pickrell K L Metzger J T Wilde N J Broadbent T R Edwards B F The use and therapeutic value of music in the hospital and operating room Plast Reconstr Surg 1950 6 02 142 152 10.1097/00006534-195008000-00005 15440335

[11] Froschauer S M Holzbauer M Kwasny O Effect of music on the efficiency of performing a microsurgical arterial anastomosis: A prospective randomized study J Hand Microsurg 2020 15 01 13 17 36761056 10.1055/s-0040-1716992 PMC9904975

[12] Shakir A Chattopadhyay A Paek L S The effects of music on microsurgical technique and performance: A motion analysis study Ann Plast Surg 2017 78(5 Suppl 4):S243 S247 28399026 10.1097/SAP.0000000000001047

[13] Moustaki M Masud D Hachach-Haram N Mohanna P N Effect of computer games and musical instruments on microsurgery J Plast Reconstr Aesthet Surg 2017 70 07 982 984 28291688 10.1016/j.bjps.2017.02.014

[14] Vouhé P R The surgeon and the musician Eur J Cardiothorac Surg 2011 39 01 1 5 21163668 10.1016/j.ejcts.2010.11.046

[15] Ullmann Y Fodor L Schwarzberg I Carmi N Ullmann A Ramon Y The sounds of music in the operating room Injury 2008 39 05 592 597 16989832 10.1016/j.injury.2006.06.021

[16] Innocenti M Malzone G Menichini G First-in-human free flap tissue reconstruction using a dedicated microsurgical robotic platform Plast Reconstr Surg 2023 151 05 1078 1082 36563175 10.1097/PRS.0000000000010108

[17] Miller R Hotton M Chan J KK Letter comments on the JPRAS paper: Training and mentorship in plastic surgery: Lessons from musical training J Plast Reconstr Aesthet Surg 2020 73 02 391 407 10.1016/j.bjps.2019.09.053 31711861

[18] Zhang W Hallock G G Empathy Arch Plast Surg 2022 49 01 3 4 35086300 10.5999/aps.2021.02271 PMC8795635

[19] Arora P Perspectives of schooling through karaoke: A metaphorical analysis Educ Philos Theory 2010 42 846 866

[20] Hallock G G Innovations: A dawning of a new age Arch Plast Surg 2021 48 02 147 148 33765730 10.5999/aps.2021.00255 PMC8007461

[21] Davidoff F Music lessons: what musicians can teach doctors (and other health professionals) Ann Intern Med 2011 154 06 426 429 21403078 10.7326/0003-4819-154-6-201103150-00009

[22] Kim D H Chandawarkar S K Krajewski A Plastic surgeon well-being, mindfulness, and the art of letting go Plast Reconstr Surg Glob Open 2025 13 01 e6440 39882436 10.1097/GOX.0000000000006440 PMC11778091

[23] Bentz M L The plastic surgeon at work and play: Surgeon health, practice stress, and work-home balance Plast Reconstr Surg Glob Open 2016 4 10 e1081 27826476 10.1097/GOX.0000000000001081 PMC5096531

[24] Bajaj A K Work/life balance: It is just plain hard Ann Plast Surg 2018 80(5S, Suppl 5):S245 S246 29596086 10.1097/SAP.0000000000001415

[25] Hong J P Hur J Work-life balance: A modern concern? Arch Plast Surg 2025 52 03 118 40386006 10.1055/a-2575-1290 PMC12081094

[26] Rohrich R J So, what do you do when the music stops? You keep dancing Plast Reconstr Surg 2017 139 01 263 264 27632400 10.1097/PRS.0000000000002903

